# Supplementary figures and images for: Beyond the VSG layer: Exploring the role of intrinsic disorder in the invariant surface glycoproteins of African trypanosomes
Source: PLoS Pathog. 2024 Apr 22;20(4):e1012186. doi: 10.1371/journal.ppat.1012186 (PMC11065263; doi:10.1371/journal.ppat.1012186)

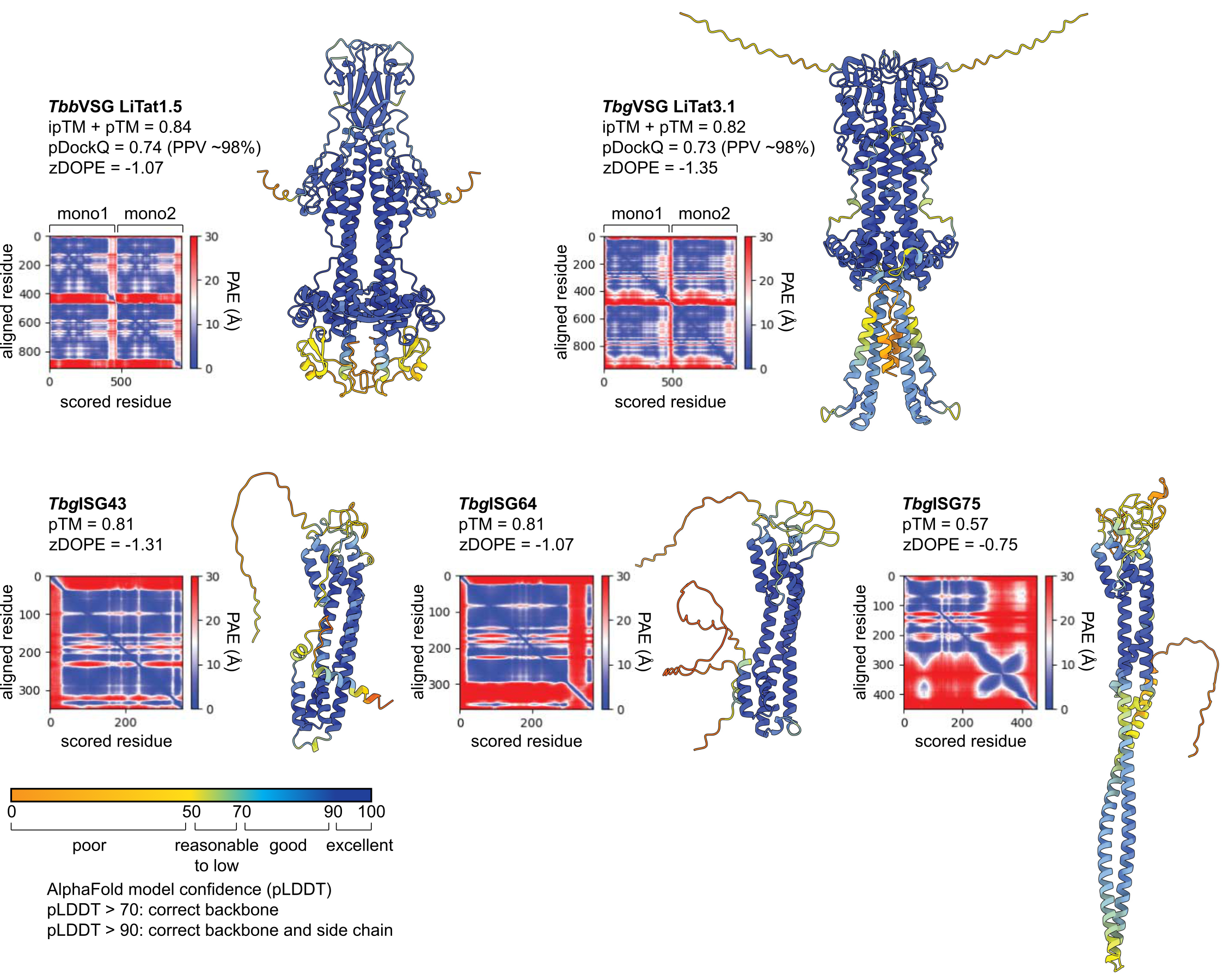

Supplement: S1 Fig — Cartoon representations of the models generated by AlphaFold2 (TbgISG43, TbgISG64, and TbgISG75) and AlphaFold-Multimer (TbbVSG LiTat1.5 and TbgVSGLiTat3.1). The models are colored according to the predicted local distance difference test (pLDDT) score, which reflects (local) model quality as indicated by the legend at the bottom. For all structures, the predicted aligned error (PAE), the normalized discrete optimized protein energy (zDOPE), and overall predicted template modeling (pTM) scores are also shown. The PAE provides a distance error for every residue pair, and is calculated for each residue x (scored residue) when the predicted and true structures are aligned on residue y (aligned residue). A zDOPE < -1 indicates that the distribution of atom pair distances in the model resembles that found in a large sample of known protein structures and that at least 80% of the model’s Cα atoms are within 3.5 Å of their correct positions. The pTM (score between 0 and 1) provides a measure of similarity between two protein structures (in this case, the predicted and unknown true structure) over all residues and thus reports on the accuracy of prediction within a single chain. For multimers, the pDockQ and AlphaFold-Multimer model confidence (0.8*ipTM + 0.2*pTM) are also shown. The interface pTM (ipTM, score between 0 and 1) provides a measure of similarity between two protein structures (in this case, the predicted and unknown true structure) over only interfacing residues and thus reports on the accuracy of prediction for a complex. The pDockQ score (between 0 and 1) is another confidence metric for protein complexes that takes into account the number of interfacing residues and their pLDDT scores. Determination of the pDockQ score can be associated to a positive predictive value (PPV), which provides an estimate for the probability that the solution is a true positive. (PNG) [file ppat.1012186.s002.png]

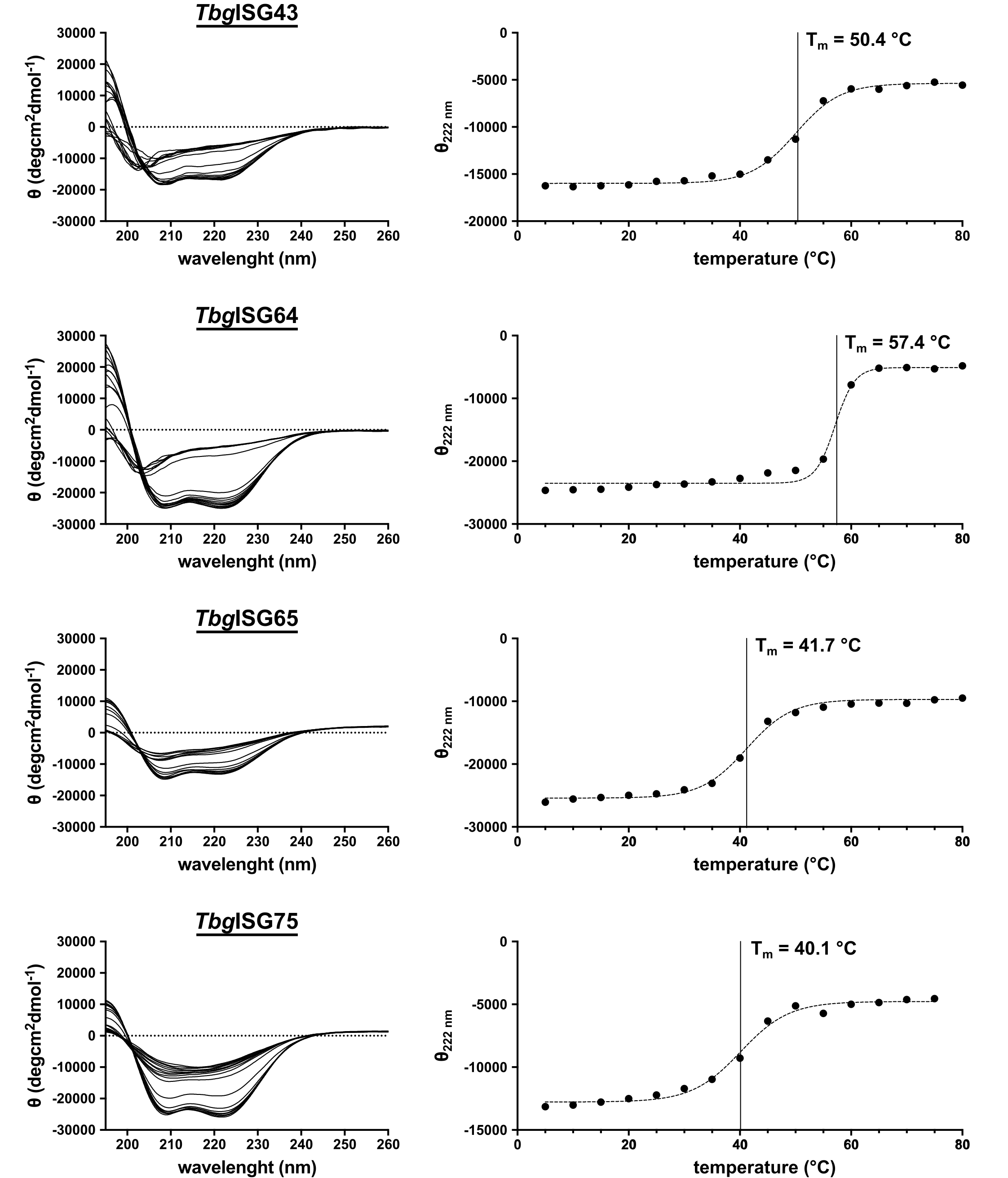

Supplement: S3 Fig — CD spectra were recorded between 5°C and 80°C in 5-degree increments. Smoothed curves show molar ellipticity from 195nm to 260nm at each temperature measured (left). Melting temperatures (Tm) were calculated from a sigmoidal curve fit to the molar ellipticity at 222nm (right). (PNG) [file ppat.1012186.s004.png]
